# Uncovering the unique characteristics of different groups of 5-HT_5A_R ligands with reference to their interaction with the target protein

**DOI:** 10.1007/s43440-024-00622-4

**Published:** 2024-07-06

**Authors:** Szymon K. Kordylewski, Ryszard Bugno, Andrzej J. Bojarski, Sabina Podlewska

**Affiliations:** Maj Institute of Pharmacology Polish Academy of Sciences, Smętna 12, 31-343 Kraków, Poland

**Keywords:** 5-HT_5A_R, Serotonin receptors, Ligand database, Docking, Molecular dynamics, Pharmacophore modeling

## Abstract

**Background:**

The serotonin 5-HT_5A_ receptor has attracted much more research attention, due to the therapeutic potential of its ligands being increasingly recognized, and the possibilities that lie ahead of these findings. There is a growing body of evidence indicating that these ligands have procognitive, pro-social, and anti-depressant properties, which offers new avenues for the development of treatments that could address socially important conditions related to the malfunctioning of the central nervous system. The aim of our study was to unravel the molecular determinants for 5-HT_5A_R ligands that govern their activity towards the receptor.

**Methods:**

In response to the need for identification of molecular determinants for 5-HT_5A_R activity, we prepared a comprehensive collection of 5-HT_5A_R ligands, carefully gathering literature and patent data. Leveraging molecular modeling techniques, such as pharmacophore hypothesis development, docking, and molecular dynamics simulations enables to gain valuable insights into the specific interactions of 5-HT_5A_R ligand groups with the receptor.

**Results:**

The obtained comprehensive set of 2160 compounds was divided into dozens of subsets, and a pharmacophore model was developed for each group. The results from the docking and molecular dynamics simulations have enabled the identification of crucial ligand–protein interactions that are essential for the compound's activity towards 5-HT_5A_R.

**Conclusions:**

The findings from the molecular modeling study provide valuable insights that can guide medicinal chemists in the development of new 5-HT_5A_R ligands. Considering the pharmacological significance of these compounds, they have the potential to become impactful treatments for individuals and communities in the future. Understanding how different crystal/cryo-EM structures of 5-HT_5A_R affect molecular modeling experiments could have major implications for future computational studies on this receptor.

**Graphical abstract:**

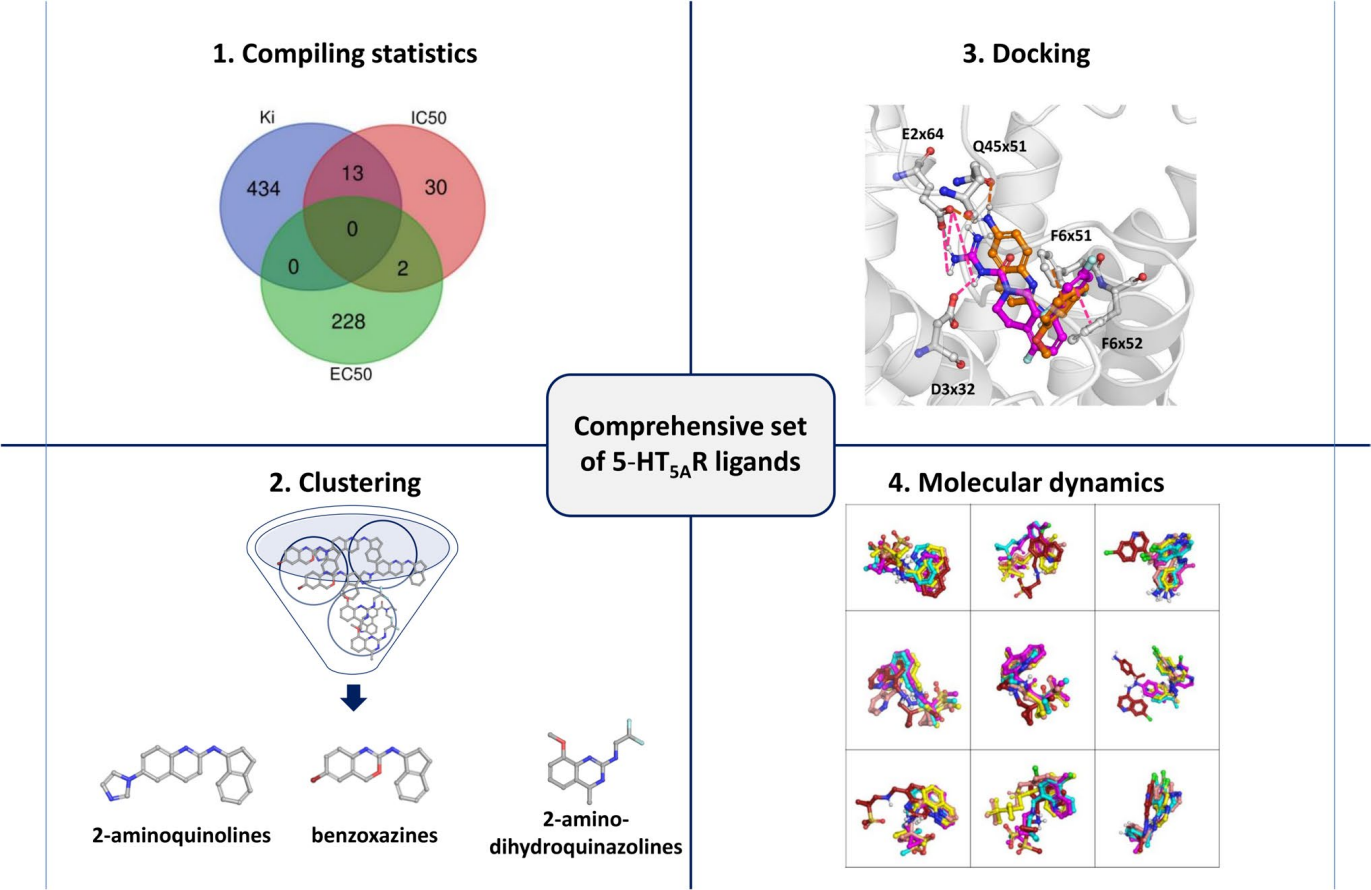

**Supplementary Information:**

The online version contains supplementary material available at 10.1007/s43440-024-00622-4.

## Introduction

The 5-hydroxytryptamine receptor 5A (5-HT_5A_R) is a G protein-coupled receptor (GPCR) belonging to the 5-HT receptor family [1, 2]. These receptors, categorized as aminergic receptors, bind to amines such as serotonin, dopamine, histamine, and norepinephrine [3, 4]. Their structural characteristic is a heptahelical domain, which consists of seven transmembrane (7TM) helices embedded in the cell membrane, allowing for communication between the extracellular environment and cytoplasmic signaling cascades and the nuclear cell-cycle machinery [5]. The 7TM domain is responsible for interacting with ligands, which trigger signal transduction and activate intracellular signaling pathways [6].

The 5-HT_5A_R was first cloned in 1992, and it is encoded by the 5-HT_5A_ gene [7, 8]. It belongs to the 5-HT_5_R subfamily, together with the 5-HT_5B_R. Although two 5-HT_5_R genes (5-HT_5A_ and 5-HT_5B_) have been described in the rodents, only 5-HT_5A_R is expressed in humans, whereas the coding sequence of 5-HT_5B_R is interrupted by stop codons, leading to a non-functional 5-HT_5B_ gene [9].

A wide distribution of 5-HT_5A_R has already been proven in the human brain, with the highest expression rate in the cortex and limbic regions [10]. Its activity is regulated by serotonin, a neurotransmitter that governs various physiological functions, such as mood, cognition, sleep, circadian rhythms, thermoregulation, pain, etc. It also has a profound impact on hemostasis, vascular tone, heart rate, respiratory drive, cell growth, and immunity [11, 12]. Recent findings on its expression patterns in regions associated with important physiological functions have opened up new avenues for exploring 5-HT_5A_R as a promising therapeutic target for psychiatric disorders [13–15]. These studies have confirmed its dysregulation in depression, anxiety, and schizophrenia, as well as its role in circadian rhythm regulation [7, 16, 17].

It has been demonstrated that 5-HT_5A_R activation initiates multiple signal transduction cascades. It is negatively coupled to adenylyl cyclase, protein kinase A, and cAMP signaling via Gα_i_/_o_ protein [18–20]. Moreover, the activation of 5-HT_5A_R transiently opens potassium channels, causing hyperpolarization of the cell membrane and a decrease in neuronal excitability [21].

The 5-HT_5A_R displays an intriguing pharmacological profile: it binds with the highest affinity to ergots, ergolines, and the synthetic agonist 5-carboxamidotryptamine (5-CT); however, most other agents acting on the 5-HT receptors lack the 5-HT_5A_R affinity [8, 22, 23]. Furthermore, the development of selective 5-HT_5A_R agents has been challenging and only a limited number of such compounds have been described so far [23, 24] (the detailed history of 5-HT_5A_R ligand discoveries can be found in the Supplementary Information, File S1). Nevertheless, the number of known 5-HT_5A_R ligands is continuously growing and the compound dataset associated with this receptor in the ChEMBL database [25] expands with each subsequent version of this repository. The growing interest in 5-HT_5A_R-oriented research is also reflected in the wealth of data available in the recently released patents, significantly expanding the collection of known 5-HT_5A_R ligands.

A growing number of data is driving the use of various statistical and machine learning approaches to uncover hidden patterns and connections within them. The swift and efficient processing of large datasets has established computational approaches as essential tools in drug design campaigns. They support drug design pipelines at every stage, starting from target identification and validation, via the detection of new potential ligands, through the optimization of their activity, physicochemical, and pharmacokinetic profile. They also play a crucial role in analyzing the outcome of in vitro and in vivo tests, results of clinical trials, and monitoring the drug effectiveness after its introduction to the market. The integration of in silico tools in the drug discovery process reduces costs and time devoted to the identification of successful drug candidates, sparking a revolution in the field of drug design [26–29].

**Fig. 1:**
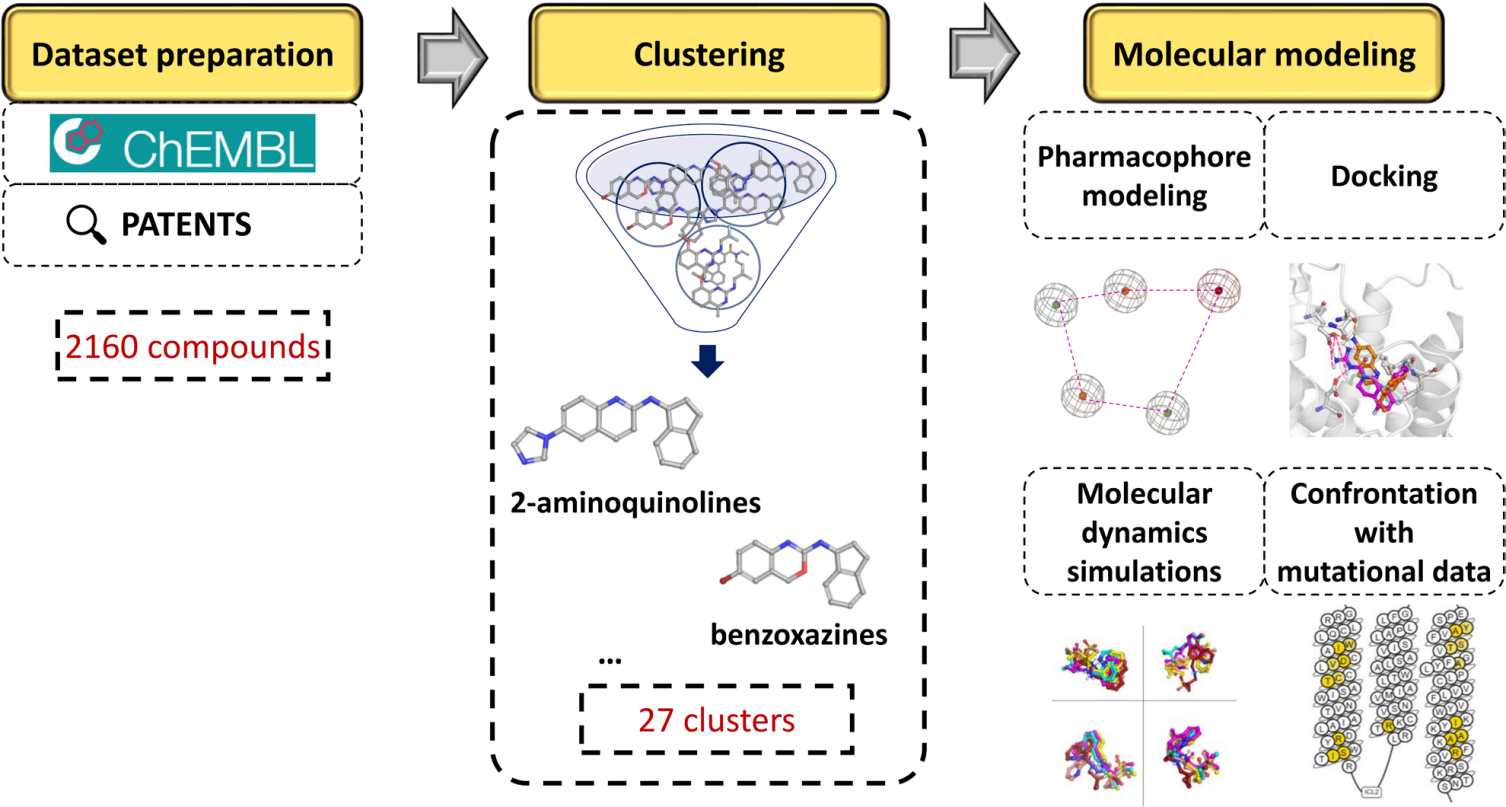
Scheme of the study

Our study thoroughly identifies molecular determinants for 5-HT_5A_R activity from ligand- and structure-based perspectives. Our work also involves the compilation of the latest information on 5-HT_5A_R ligands present in both literature and patent data. The manually extracted information from patents (of several hundreds of records) greatly enhanced the ChEMBL-based dataset, resulting in a valuable collection of 5-HT_5A_R ligands. This resource is of great importance to the whole medicinal chemistry community, significantly supporting further research within this target (we share all data via the Supplementary Information, File S2). We analyzed the dataset of 5-HT_5A_R agents globally, but we also focused on particular chemical groups to gain a comprehensive understanding of the activity profile of particular compounds. By employing pharmacophore modeling, docking, and molecular dynamics (MD) simulations, we were able to gain a broad understanding of the activity profile of specific compounds and their ligand-receptor contacts using the recently released crystal/cryo-EM structures of this receptor [23, 30]. The comprehensive compilation, analysis, and comparison of existing ligands, along with a detailed examination of ligand-receptor contacts, will undoubtedly aid in developing new drug candidates displaying activity towards 5-HT_5A_R. The scheme of the whole study is presented in Fig. 1.

## Materials and methods

At first, all records referring to the human 5-HT_5A_R were retrieved from the ChEMBL database version 33 (Target ChEMBLID: CHEMBL3426) [25]. Such a compound database was then enriched with the manually extracted patent data. The resulting dataset was subjected to manual clustering aided by the results of the automated grouping carried out in the Canvas module from the Schrödinger Suite 2023 [31]. For each compound group with at least ten ligands, the pharmacophore model was developed using Phase [32]. The compounds were docked to the available crystal structures of 5-HT_5A_R (7UM4 [23], 7UM5 [23], 7UM6 [23], 7UM7 [23]; 7X5H [30] was not considered due to crystallization with the same ligand as in 7UM5, and 7UM5 had better resolution). Docking was performed in two modes: separately for particular clusters (those with the highest number of examples) and collectively using the ChEMBL-based dataset. Due to a lack of information about the inactive compounds in patents, the collective analysis was limited to data included in the ChEMBL database. Glide [33] in extra precision mode was used for docking, with compounds prepared using LigPrep [34] from the Schrödinger Suite (protonation states generated at pH 7.4 ± 0.0, and all possible stereoisomers were enumerated; other settings remained at default). The reliability of docking was verified by re-docking the co-crystallized ligands and examination of the RMSD of the obtained conformations with reference to the co-crystallized pose. The resulting ligand-receptor complexes were encoded in the form of the structural interaction fingerprint (SIFt) [35], and the contact frequencies for a particular group of compounds were measured. Results obtained via the statistical analysis of docking were confronted with the mutagenetic data reported for the 5-HT_5A_R (GPCRdb [36] data were used). To gain a detailed understanding of the ligand–protein contacts in the compound complexes with 5-HT_5A_R for different crystal structures, a series of MD simulations was carried out for the selected set of quinoline derivatives (CHEMBL5077128, CHEMBL5082447, CHEMBL2005743), starting from the ligand-receptor complexes obtained in docking. To capture differences among the available crystal structures, only EC_50_ data were taken into account, as there is only one crystal structure available for 5-HT_5A_R in its inactive conformation. The MD simulations were carried out in Desmond [37], using TIP3P solvent model [38] and POPC (palmitoyl-oleil-phosphatidylcholine) as a membrane model and OPLS3e force-field under the pressure of 1.01325 bar and in the temperature of 300 K. The box shape was orthorhombic with dimensions of 10 Å × 10 Å × 10 Å. In each case, the system was neutralized by the addition of the respective number of Cl- ions and relaxed before the simulation; the duration of each simulation was equal to 1000 ns. At first, the results were analyzed in terms of the stability of the compound pose in the binding pocket. The assessment was carried out qualitatively through visual inspection of the compound orientations at specific time steps of the simulation (starting pose, after 250 ns, after 500 ns, after 750 ns, and after 1000 ns) and quantitatively by examining the compound's RMSF during the simulation. Finally, correlational studies were conducted to identify the positions with the highest correlation (expressed by Spearman’s correlation coefficient) between the frequency of compound interaction with particular amino acids and the outcome of the experimental verification of compound activity.

## Results

### Analysis of ChEMBL data

**Fig. 2:**
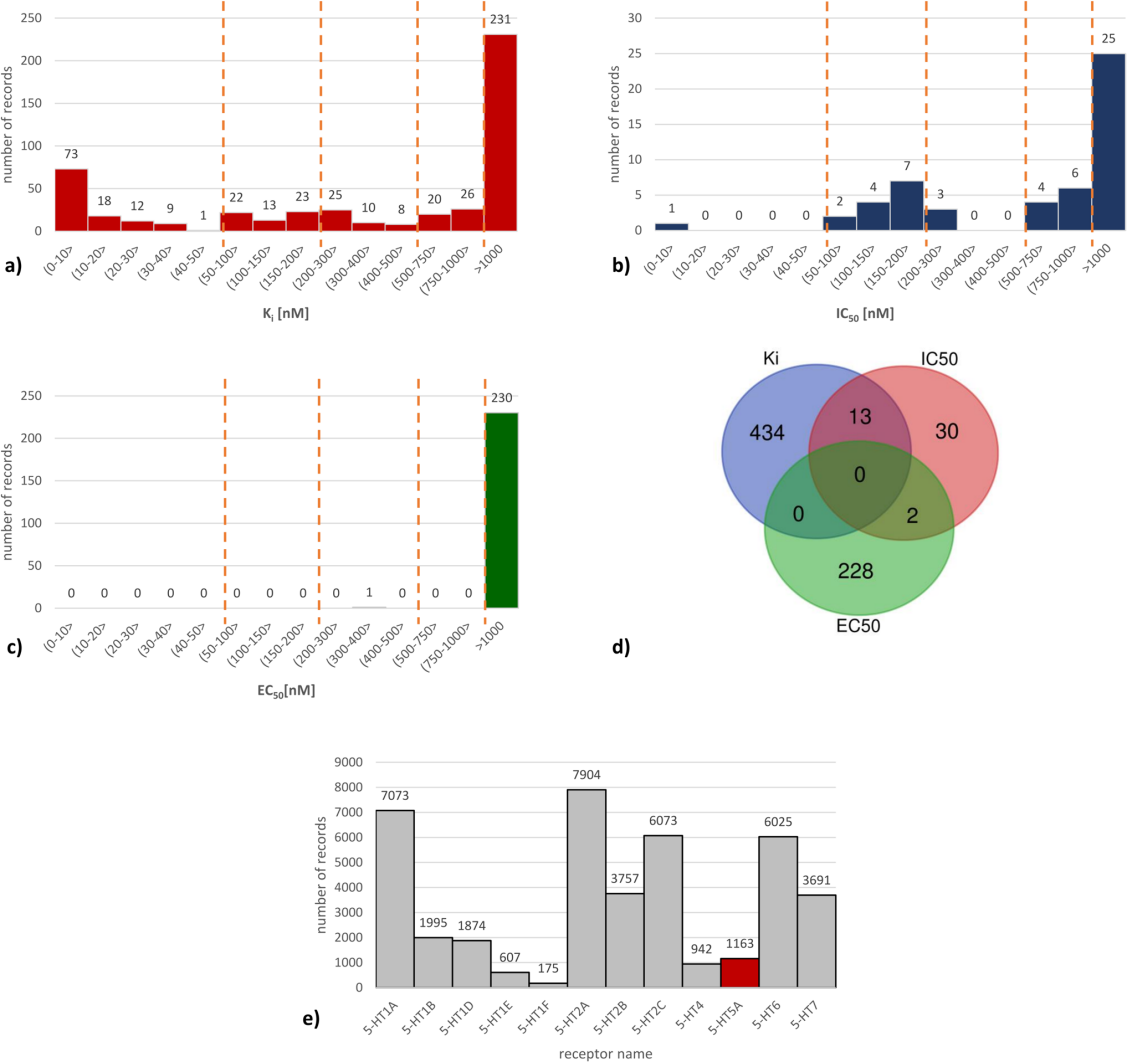
Distribution of activity parameters values of 5-HT_5A_R ligands deposited in the ChEMBL database (orange lines refer to step changes in the scale), **a** K_i_, **b** IC_50_, **c** EC_50_, **d** Venn diagram for particular parameters values determined for 5-HT_5A_R ligands, created on-line at https://bioinformatics.psb.ugent.be/webtools/Venn/; **e** the number of records present in the ChEMBL database referring to different serotonin receptor subtypes

The distribution of activity parameter values for ChEMBL records referring to human 5-HT_5A_R is presented in Fig. 2.

Overall, there are 491 records with K_i_ values reported (Fig. 2a), 231 of them related to EC_50_ (Fig. 2c), and only 52 providing information about the IC_50_ values of 5-HT_5A_R ligands (Fig. 2b). These refer to 447 unique compounds with determined K_i_ values, 230 unique ligands with known EC_50_ values, and 45 with known IC_50_ values (Fig. 2d). Among these compounds, 135 have K_i_ values below 100 nM, and 3 compounds have IC_50_ values below 100 nM. Interestingly, for all ligands with EC_50_ values determined, they exceeded 100 nM, with the most active compound having an EC_50_ value of 354.8 nM).

When comparing the number of identified 5-HT_5A_R ligands to other related targets (Fig. 2), we find that it is significantly lower than the highest populated in ligands 5-HTR subtypes. The ChEMBL database currently contains only 1163 records for this receptor subtype, which indicates the need for further research and development in this area. In contrast, the number of records for 5-HT_2A_R and 5-HT_1A_R are almost 8000 (7904) and 7073, respectively. The other receptor subtypes with a high number of ligands include 5-HT_2C_R, 5-HT_2B_R, 5-HT_6_R, and 5-HT_7_R. However, some subtypes have relatively lower amounts of data, such as 5-HT_1E_R (607 records), 5-HT_1F_R (175 records), and 5-HT_4_R (942 records).

The ChEMBL-based compound database prepared within the study was also enriched with the manually extracted patent data, providing several hundreds of additional data points thoroughly described in further sections of the article and shared via the Supplementary Information. Such a huge amount of information on 5-HT_5A_R in patents is a promising sign of a growing interest in this target by pharmaceutical companies and suggests potential therapeutic applications of its ligands.

### Clustering and characterization of different groups of 5-HT_5A_R ligands

**Fig. 3:**
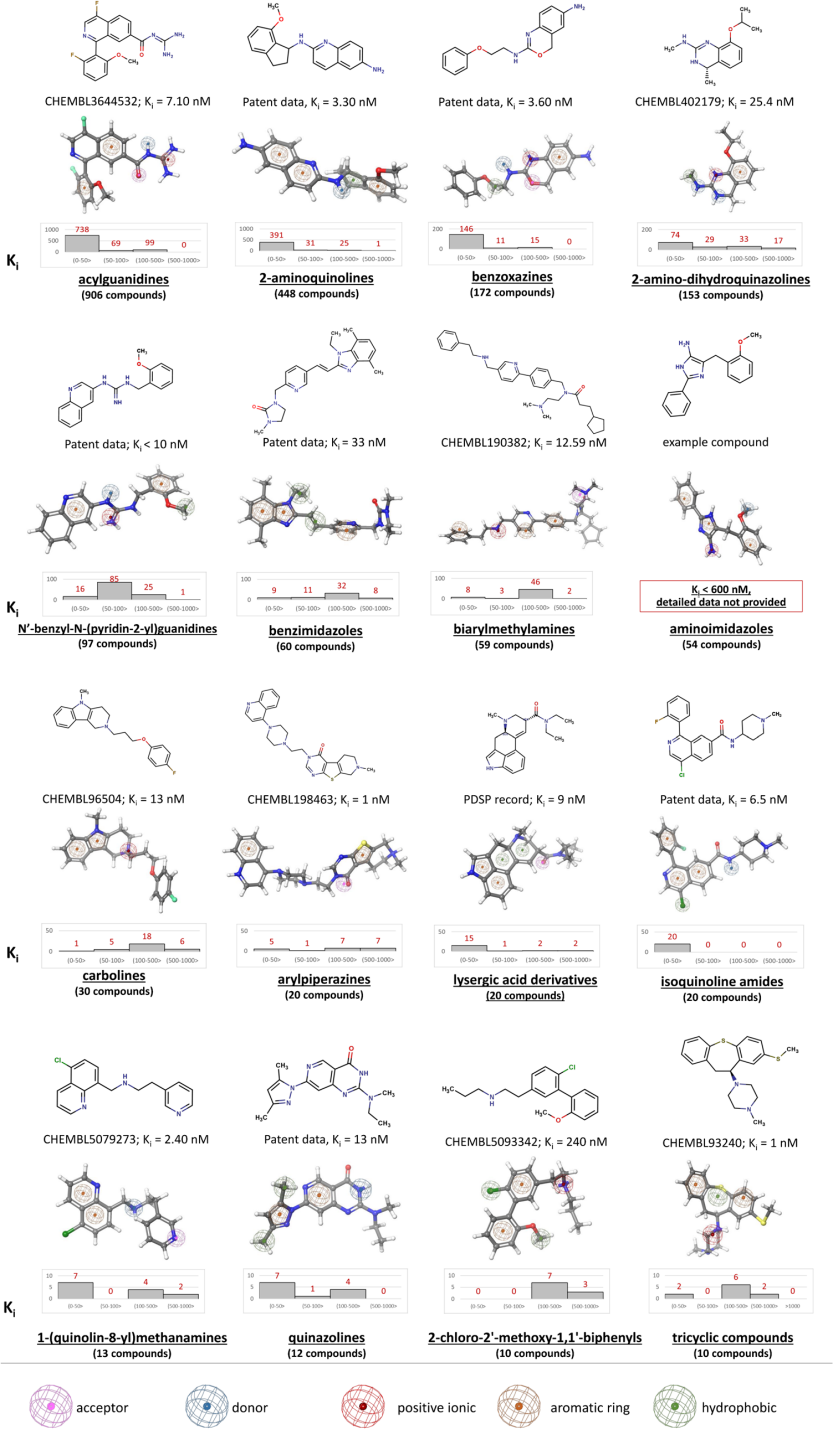
The most populated clusters of known 5-HT_5A_R ligands together with the distribution of the K_i_ values within each cluster and pharmacophore model developed for each group of compounds

**Fig. 4:**
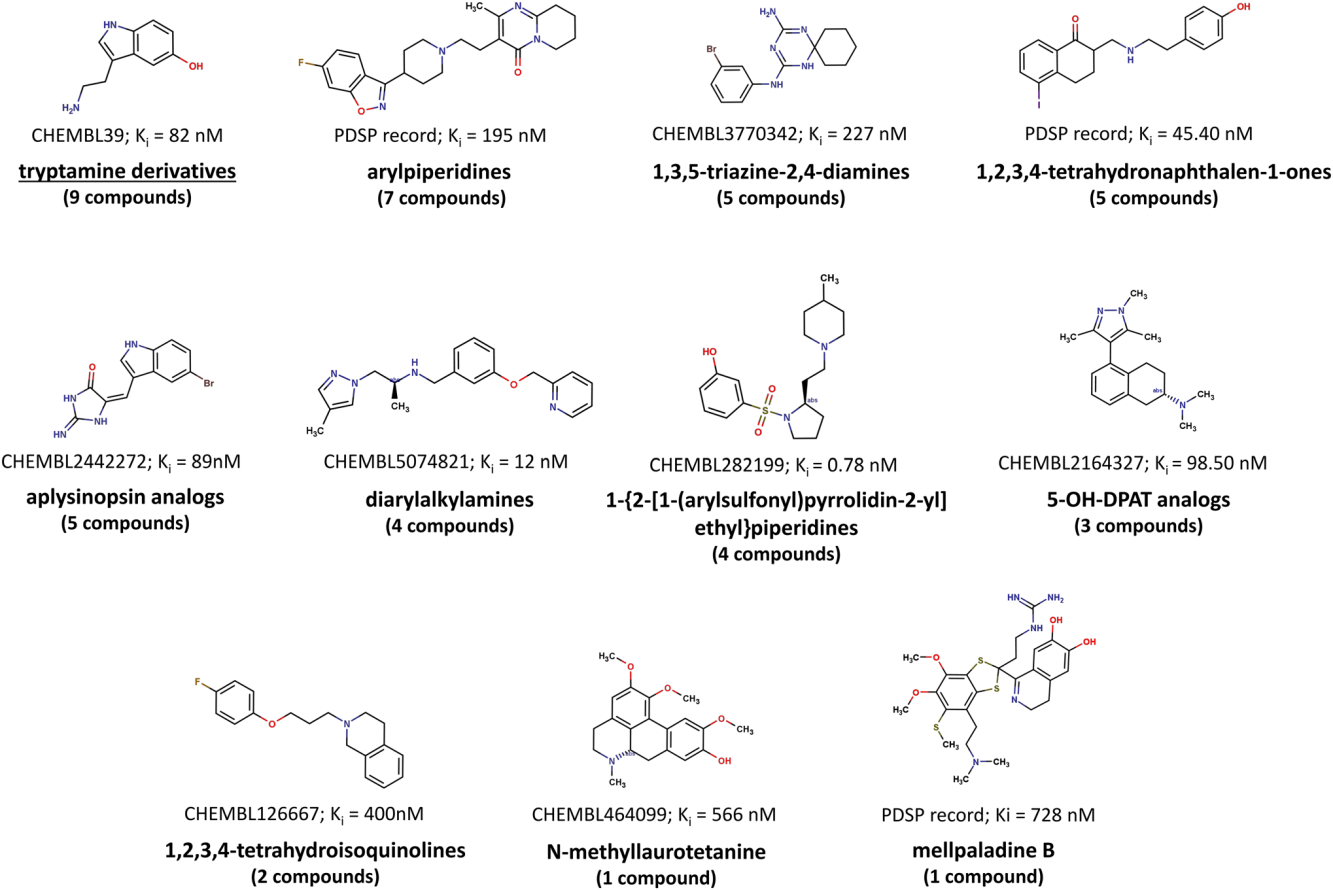
Examples of less populated groups of 5-HT_5A_R ligands

Groups of 5-HT_5A_R ligands organized on the basis of their chemical structure are summarized in Figs. 3 and 4. The latter presentation also encompasses the distribution of K_i_ values associated with the specific cluster and pharmacophore model developed for each group.

The largest chemical class of known 5-HT_5A_R binders (as presented in Fig. 3) consists of acylguanidines with over 900 compounds reported [39–43]. It is worth pointing out that these compounds display high 5-HT_5A_R affinity, with the great majority of them having K_i_ values below 50 nM. Another significant chemical class of 5-HT_5A_R ligands includes 2-aminoquinolines (448) [44–51] and benzoxazines (172) [52, 53], many of which also have a significant number of compounds with K_i_ values towards 5-HT_5A_R below 50 nM (391 and 146, respectively). It is worth mentioning that these two classes of 5-HT_5A_R ligands are not present in the ChEMBL database; they are covered in patents. Among 5-HT_5A_R ligands, there are also 153 2-amino-dihydroquinazolines [54–58] with high-to-moderate affinity to the receptor, 97 N’-benzyl-N-(pyridin-2-yl)guanidines [59], 90 benzimidazoles [60], 59 biarylmethylamines [61], and 54 aminoimidazoles. The exact values of activity parameters are not provided for the aminoimidazoles, but the information included in the patent indicates that K_i_ values towards 5-HT_5A_R for the whole group of compounds are below 600 nM. There is also a group of 30 carbolines with moderate 5-HT_5A_R affinity [62, 63]. Additionally, there are three sets of 20-membered compound groups of ligands, namely arylpiperazines (ChEMBL data), lysergic acid derivatives (PDSP data), and isoquinoline amides (patented) [64]. Moreover, the ChEMBL database contains information about 13 1‐(quinolin‐8‐yl)methanamines [24], 12 quinazolines, 10 tricyclic compounds, and 10 2‐chloro‐2'‐methoxy‐1,1'‐biphenyls.

In addition, there are over a dozen groups of 5-HT_5A_R ligands with only a few example compounds reported so far (Fig. 4). The PDSP database contains arylpiperidines, 1,2,3,4-tetrahydronaphthalen-1-one- and mellpalladine B-based 5-HT_5A_R ligands, whereas in the ChEMBL database, we can find additionally tryptamines, 1,3,5-triazine-2,4-diamines, aplysinopsin analogs, diarylalkylamines, 1‐{2‐[1‐(arylsulfonyl)pyrrolidin‐2‐yl]ethyl}piperidines, 5-OH-DPAT analogs, 1,2,3,4-tetrahydroisoquinolines, and one N‑methyllaurotetanine-based ligand.

### Docking

In 2022, significant progress was achieved in comprehending the crystal structures of the 5-HT_5A_R. As a result of extensive research, two papers revealed a total of 5 crystal/cryo-EM structures of this receptor [23, 30]. These findings have been instrumental in advancing our understanding of the receptor and its role in biological processes. When developing new ligands for a specific biological target through structure-based drug design, understanding the spatial structure of a protein is critical. This knowledge enhances the reliability of docking studies and paves the way for the development of new effective ligands.

**Fig. 5:**
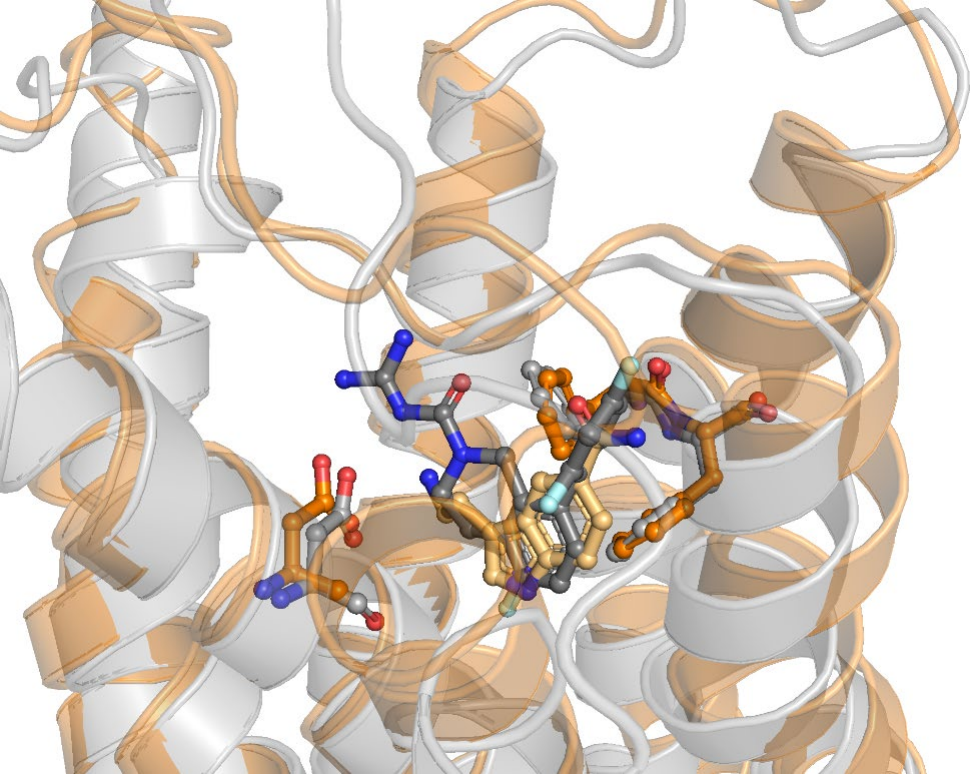
Visualization of example 5-HT_5A_R crystal structures: 4UM4 (gray), and 4UM5 (orange)

The available crystal structures of the 5-HT_5A_ receptor are summarized in Table 1 and the example structures with the co-crystallized ligands are presented in Fig. 5.

**Table 1:** The summary of the available crystal structures of 5-HT_5A_R

PDBID	Receptor state	Method of structure determination	Resolution [Å]	Co-crystallized ligand	Structure of the co-crystallized ligand
7X5H	Active	Cryo-EM	3.1	5-CT (agonist)	
7UM4	Inactive	X-ray	2.8	CHEMBL3654198 (antagonist)	
7UM5	Active	Cryo-EM	2.7	5-CT (agonist)	
7UM6	Active	Cryo-EM	2.8	Lisuride (agonist)	
7UM7	Active	Cryo-EM	2.8	Methylergonovine (agonist)

The reliability of docking studies was confirmed through the re-docking of the co-crystallized ligands. The RMSD values between the compound pose obtained in docking and the co-crystallized conformation were as follows: 0.205 Å, 0.041Å, 0.224 Å, and 0.283Å for 7UM4, 7UM5, 7UM6, and 7UM7 crystal structures, respectively.

**Fig. 6:**
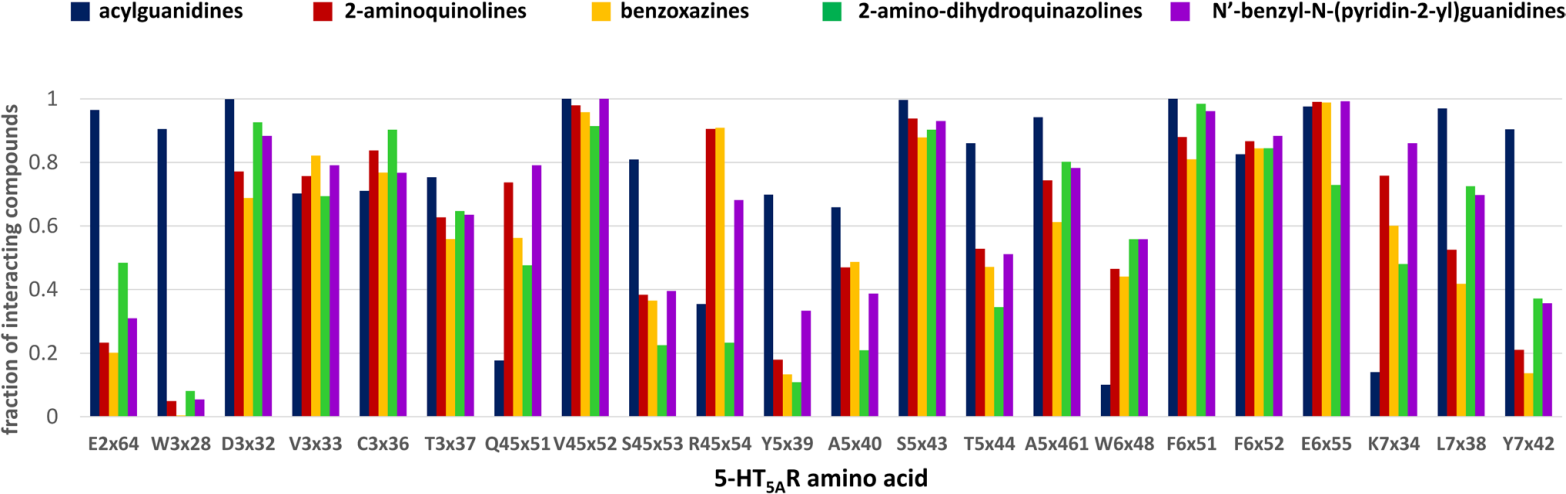
The interaction frequency of the most populated clusters of 5-HT_5A_R ligands with the 7UM4 crystal structure (positions making contact with more than 60% of compounds from at least one of the considered groups are presented)

**Fig. 7:**
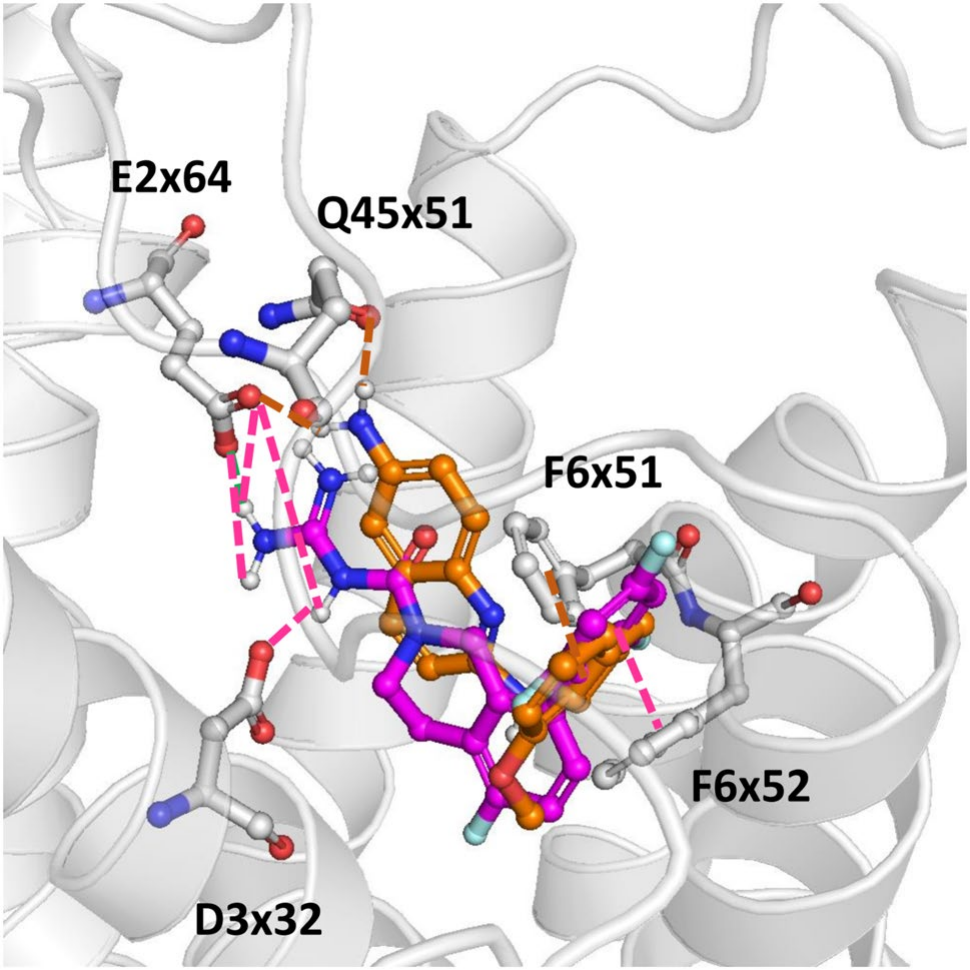
Docking poses of representatives of 2-aminoquinolines (orange) and acylguanidines (magenta) of structures presented in Fig. 3

The docking study outcomes were initially analyzed in a cluster-based manner, focusing on the most populated clusters of 5-HT_5A_R ligands. Such an approach enabled the indication of the amino acids that most frequently interact with particular groups of compounds (Fig. 6). For example, it revealed the consistent contact of all the analyzed groups of compounds with amino acids such as D3x32, V3x33, C3x36, V45x52, S5x43, A5x461, F6x51, F6x52, and E6x55. On the other hand, there is a noticeable consequent contact pattern for acylguanidines, which is not so typical for the other considered groups of 5-HT_5A_R ligands. For example, almost all compounds from this group make interact with E2x64, W3x28, S45x53, T5x44, L7x38, and Y7x42, which is not so frequent for 2-aminoquinolines, benzoxazines, 2-amino-dihydroquinazolines, and N’-benzyl-N-(pyridin-2-yl)guanidines. Docking results to 7UM4 of representatives of 2-aminoquinolines and acylguanidines (compounds presented in Fig. 3) are visualized in Fig. 7. Although both compounds display very high 5-HT_5A_R affinity (K_i_ = 3.30 nM and K_i_ = 0.68 nM for 2-aminoquinolines acylguanidine example, respectively) and occupy a similar area of the 5-HT_5A_R binding pocket, their orientation in the binding site, as well as the ligand–protein contacts formed are slightly different. In particular, both compounds form π-π contacts with the phenylalanine cluster (F6x51 and F6x52) and hydrogen bonds with E2x64; however, CHEMBL3654198 with the acylguanidine core makes also several hydrogen bonds with D3x32 whereas the 2-aminoquinoline-based ligand forms a hydrogen bond with Q45x51.

**Fig. 8:**
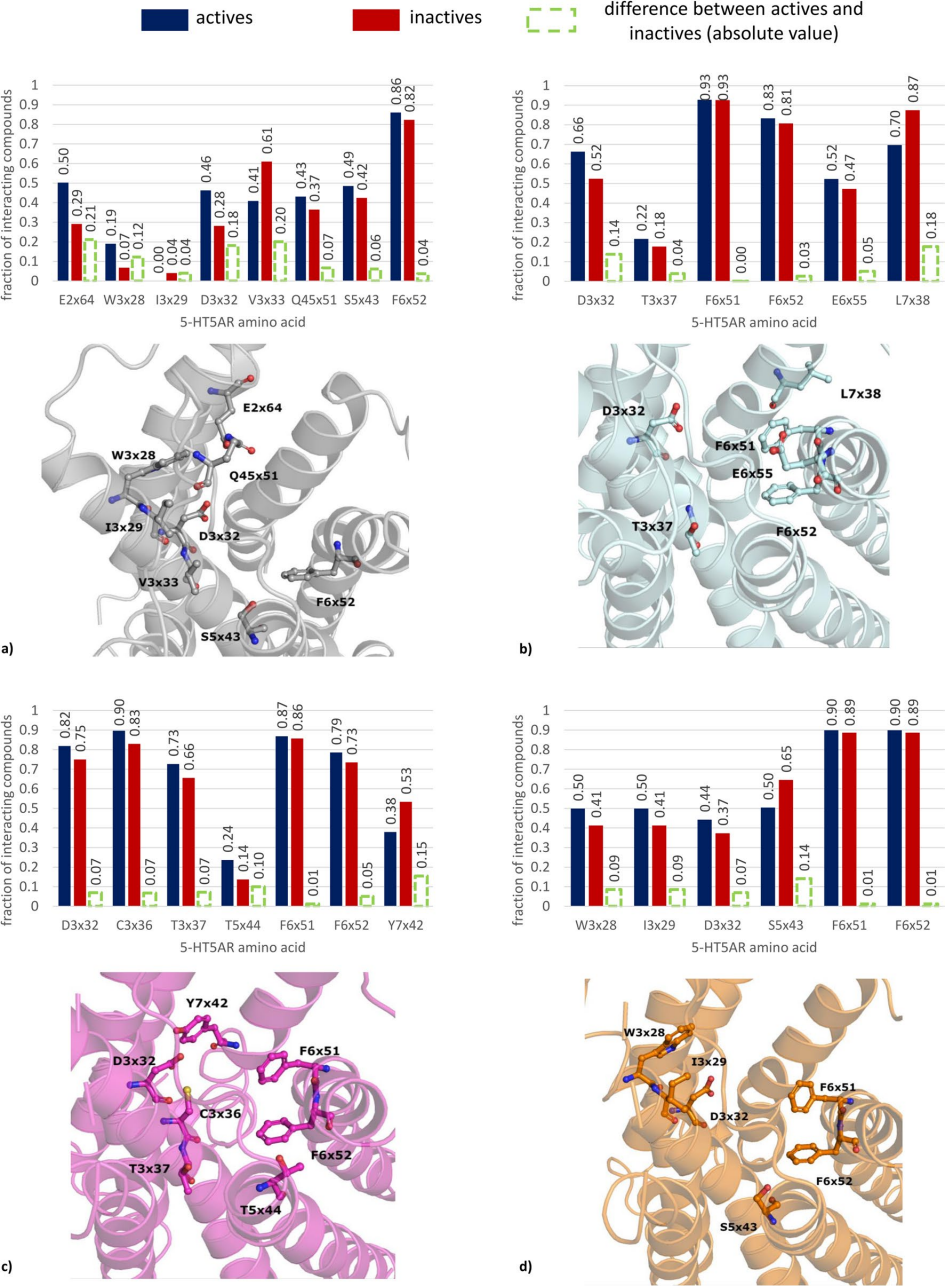
Amino acids with the highest difference in the ligand–protein interaction frequency between active (K_i_ < 1000 nM) and inactive (K_i_ > 1000 nM) compounds observed for different 5-HT_5A_R crystal structures: **a** 7UM4, **b** 7UM5, **c** 7UM6, **d** 7UM7; amino acid numbering according to the GPCRdb scheme

When analyzing the docking studies outcome globally, the 5-HT_5A_R ligands extracted from the ChEMBL database (only ChEMBL data were used due to the biased activity distribution in patents) were categorized into active/inactive groups based on their K_i_ values: compounds with K_i_ values below 1000 nM were classified as active, while those with K_i_ values above 1000 nM were considered inactive.. The absolute difference between the fraction of interacting active compounds and the fraction of interacting inactive compounds was determined. Positions with the highest difference in interaction contact frequency between the two groups of compounds were indicated in Fig. 8.

In general, the results are slightly different when various 5-HT_5A_R crystal structures are considered, although in general, the indicated positions align with the crucial amino acids reported for other 5-HT receptor subtypes [65–80]. For example, the consistent variance in interaction frequency between active and inactive compounds across different crystal structures highlights the importance of the aspartic acid located in the third transmembrane helix (D3 × 32 according to the GPCRdb numbering). Still, only for 7UM4 (referring to the inactive conformation of 5-HT_5A_R) and 7UM5 (reflecting the active state of 5-HT_5A_R) the difference exceeded 10%, being equal to 18% and 14%, respectively. In contrast, for 7UM6 and 7UM7, the interaction rates of active and inactive compounds with D3x32 exhibited a more similar pattern, with differences of 7% for both crystal structures. Another position that was consistently identified for its discriminative power between active and inactive compounds was F6x52, although, for none of the studied crystal structures, the difference exceeded 5%.

Overall, the highest discriminative power was observed for the 7UM4 crystal structure with 3 positions (E2x64, D3x32, V3x33) showing active/inactive contact frequency difference exceeding 15% (21%, 18%, and 20%, respectively). Also for 7UM4 there was the highest total number of indicated positions (8). The crystal structures 7UM5 and 7UM6 displayed the highest discriminative power within the 7th transmembrane helix with differences of 18% for L7x38 in 7UM5 and 15% for Y7x42 in 7UM6. On the other hand, S5x43 was identified as the most discriminative position in the 7UM7-based studies.

**Fig. 9:**
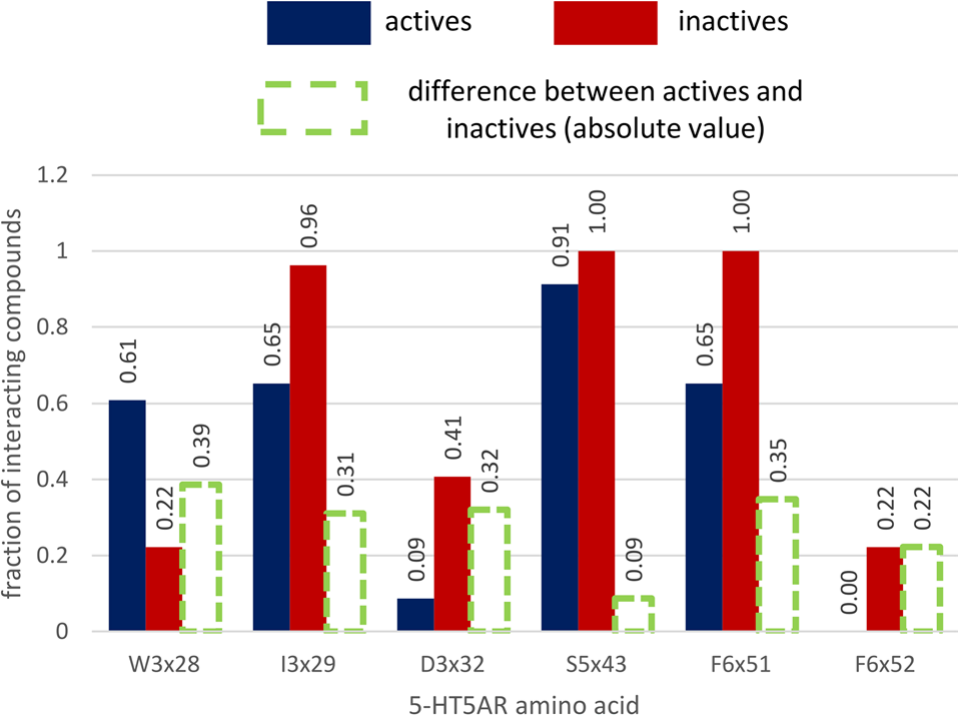
Amino acids with the highest difference in the ligand–protein interaction frequency between 5-HT_5A_R antagonists (IC_50_ < 1000 nM) and inactive compounds (IC_50_ > 1000 nM)

The docking data were also analyzed for the functional experiments. At first, the potency of compounds to inhibit 5-HT_5A_R (IC_50_ data) was taken into account. As this parameter expresses the compound’s potency to inhibit the receptor, only its inactive conformation was considered (the 7UM4 crystal structure (Fig. 9)). The most evident observation regarding this data is that the indicated differences between the contact frequency of the confirmed 5-HT_5A_R antagonists and non-antagonists were much higher than in the K_i_-based analysis. There are four positions (W3x28, I3x29, D3x32, and F6x51) for which the difference between the contact frequency of the two groups of compounds exceeded 30%. Qualitatively, the positions identified in the IC_50_-based studies align with those from the K_i_-based experiments.

**Fig. 10:**
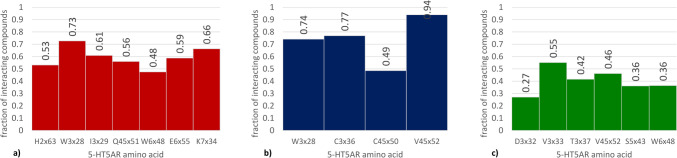
Amino acids with the highest difference in the ligand–protein interaction frequency between 5-HT_5A_R agonist (EC_50_ < 1000 nM) and inactive compounds (EC_50_ > 1000 nM) for different crystal structures: **a** 7UM5, **b** 7UM6, **c** 7UM7

The analysis of the EC_50_ data was carried out in an analogous manner with some unique considerations due to the presence of only one active compound (Fig. 10). Figure 10 presents the positions for which the highest interaction frequency difference was observed, but with the assumption that particular contact did not occur for the examined agonist. Interestingly, for 7UM5- and 7UM6-based experiments, indeed the D3x32 position was not indicated, but for 7UM7-based studies, it is present on the diagram, which means that the one known 5-HT_5A_R agonist does not make contact with this residue when docked to 7UM7. Also, the phenylalanines from the 6th TM helix (F6x51 and F6x52) are not present in this comparison (as they make contact also with the active compound), but there is also a set of residues which were indicated in the previous analysis (Fig. 8), but they are not forming interaction with the examined agonist, such as W3x28, W6x48, V3x33, etc.

### Molecular dynamics simulations

**Fig. 11:**
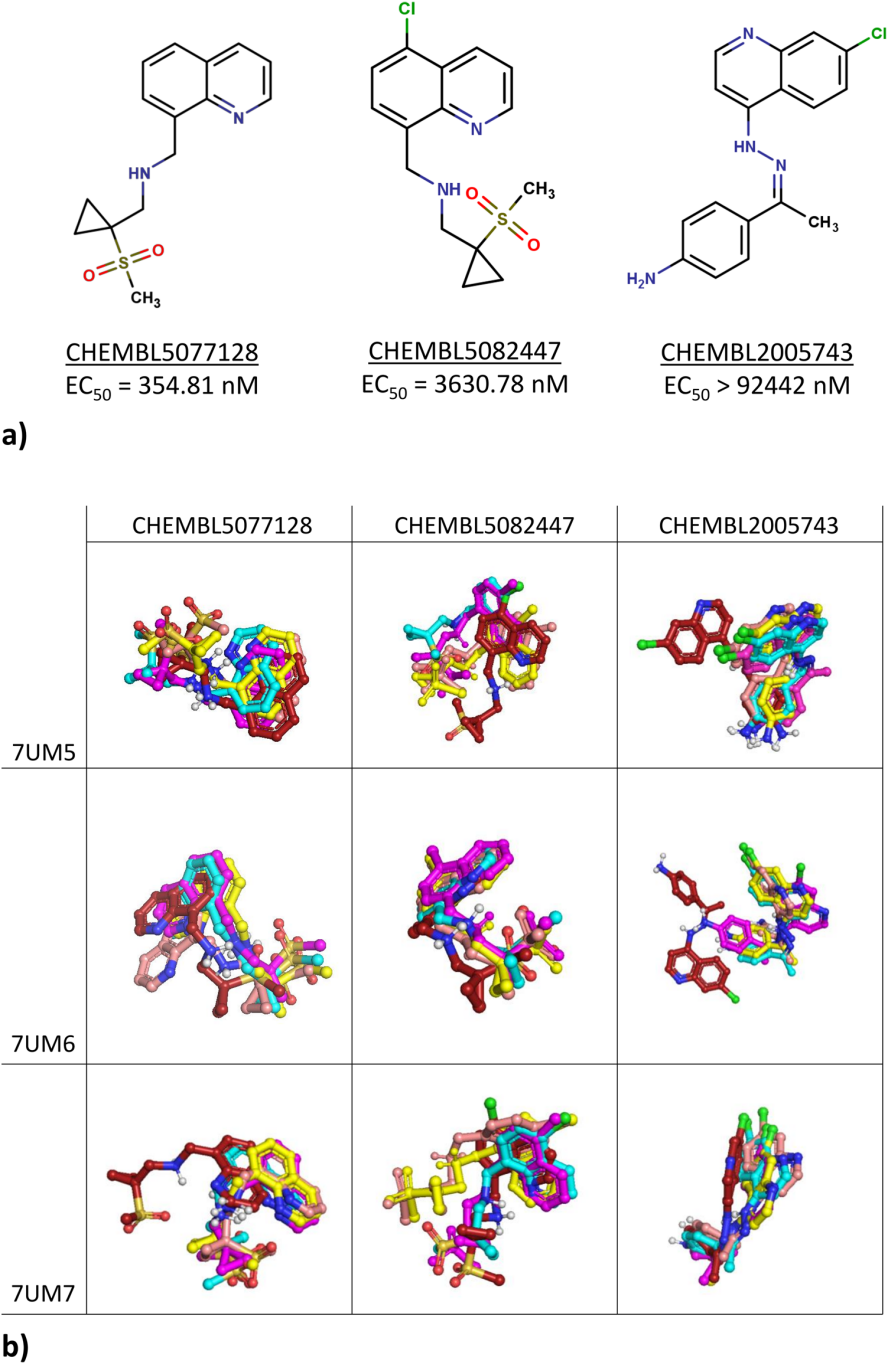
**a** Structures and activities of compounds examined in the molecular dynamics simulation studies, **b** selected compound conformations obtained in the MD for different time steps of the simulation: firebrick—starting conformation, cyan: 250 ns, magenta: 500 ns, yellow: 750 ns, salmon: 1000 ns.

The structures of ligands that underwent MD simulations are presented in Fig. 11. The figure indicates that the inactive CHEMBL2005743 did not preserve the obtained docking pose and in the initial time of the simulation its conformation changed. On the other hand, the outcome of the 7UM7-based studies contrasts with these results. In this case, CHEMBL2005743 was found to be most stably fit in the 5-HT_5A_R binding pocket, whereas the most active CHEMBL5077128 changed its initial pose and remained in the new conformation until the end of the simulation.

The MD data were also analyzed more formally by the analysis of compound RMSF (Table 2).

**Table 2:** Analysis of the average ligand RMSF during MD simulations with different crystal structures

Ligand/crystal structure	CHEMBL5077128	CHEMBL5082447	CHEMBL2005743
7UM5	0.284	1.143	0.284
7UM6	0.309	0.518	0.566
7UM7	0.858	0.830	0.364

The data provided in Table 2 revealed that although in the case of the 7UM5-based studies the inactive CHEMBL2005743 initially underwent a significant conformational change, but then stabilized in the subsequent frames of the simulation, with an average RMSF similar to that of the most active CHEMBL5077128. On the other hand, the conformation of CHEMBL5082447 varied significantly in the 5-HT_5A_R binding site when the 7UM5 crystal structure was taken for studies. The only crystal structure for which the activity-RMSF relationship for the examined compounds was preserved was 7UM6. However, before making the general recommendation for its usage for compound EC_50_ prediction, more extensive studies should be carried out, which is not possible at this moment due to the limited availability of agonistic structures reported so far.

Finally, the correlational studies between the frequency of compound interaction with particular amino acids were carried out to pinpoint positions with the highest Spearman’s correlation coefficient with the experimental activity verification results. This analysis led to the identification of the set of positions for which the correlation was equal to ± 1 (Table 3).

**Table 3:** Amino acids interactions which corresponded to the highest extent with the EC_50_ experimental values

Crystal structure/correlation	7UM5	7UM6	7UM7
+ 1	V3x33, I4x56, S4x57, P4x60, L4x61, E5x36, A5x40, S5x43	V3x33, E6x55	I3x29, D3x32, C3x36, W6x48, L7x38, Y7x42
− 1	W3x28, I3x29, C45x50, Q45x51, V45x52, F6x51, L7x38	D3x32, W6x48, F6x51, F6x52, L7x38, Y7x42	V3x33, Y5x39, A5x40, F6x52

### Confrontation with mutagenetic data

**Fig. 12:**
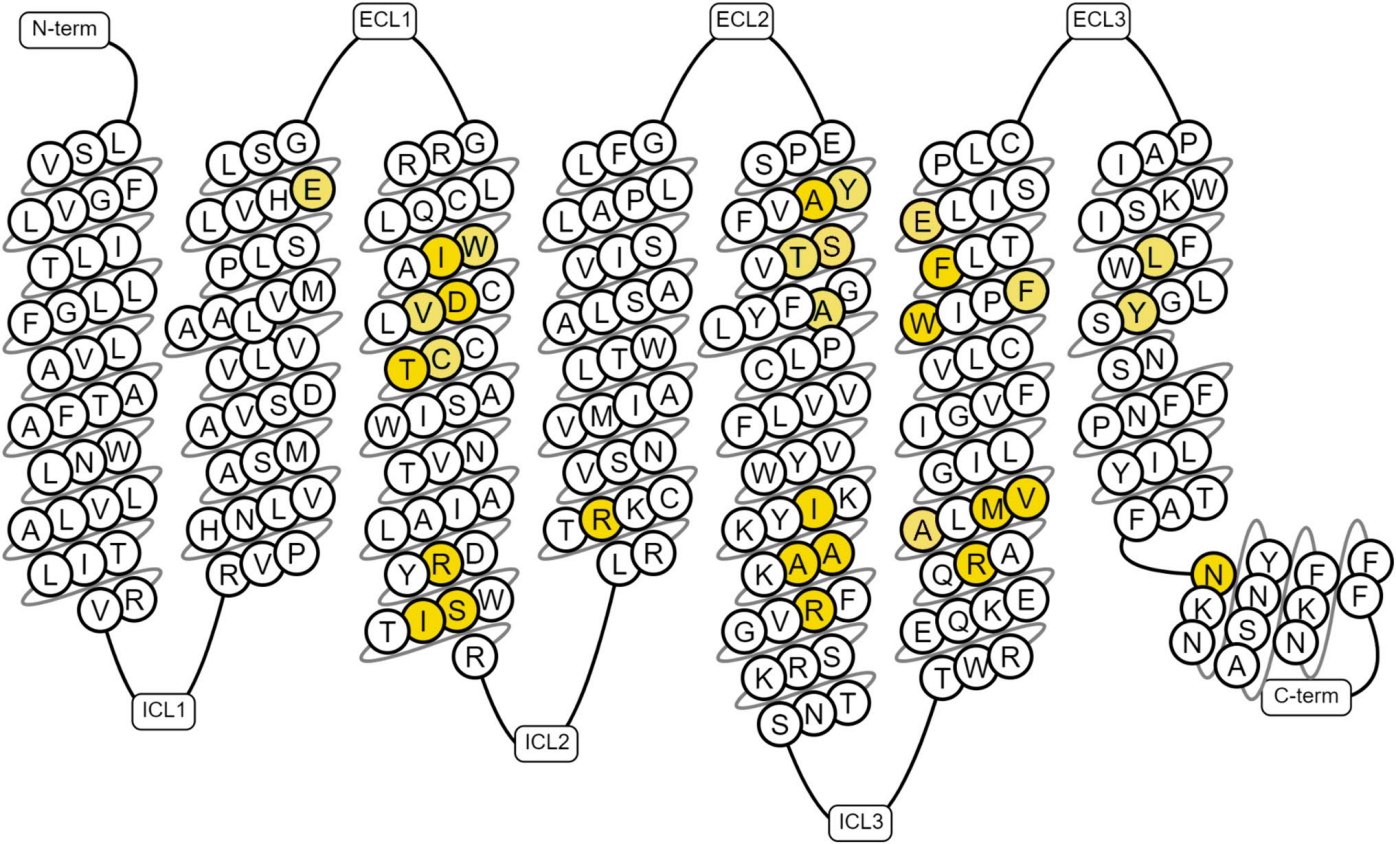
Mutagenetic data available for the 5-HT_5A_R; figure created with the use of the GPCRdb service [36]; yellow color indicates change of the ligand binding affinity upon particular residue mutation

Results obtained via the computational studies were confronted with the mutagenetic data reported for the 5-HT_5A_R (visualization prepared in the GPCRdb service, Fig. 12). The list of amino acids indicated both in docking and MD simulations were consistent with the experimental outcome of the mutagenesis experiments, although there are variations in the observed effect of amino acid substitution (increase/decrease of a compound potency).

There is clear evidence supporting the importance of the D3x32 for ligand binding, as all available data report the activity loss of a ligand upon the substitution of this amino acid by alanine [18, 25]. Both papers also report the activity change upon the F6x52A mutation; however, this effect is much less pronounced compared to the D3x32 substitution, as it is approx. 1.5-fold decrease in activity related to this mutation. E2x64 was the position for which high discriminative power was indicated for the 7UM4-based studies. The effect of the mutation at this position strongly depended on the substituted amino acid and the specific ligand being examined [23]. For example, the E2x64D mutation led to a slight increase in the activity of CHEMBL3654198, whereas the E2x64 mutation to glutamine or alanine led to a significant decrease of this ligand affinity to 5-HT_5A_R. On the other hand, when lysergide was used as the examined ligand, all E2x64D, E2x64Q, and E2x64A substitutions resulted in the worsening of the ligand affinity. Another position with high discriminative power for 7UM4-based docking experiments was V3x33 (20% of the difference between the contact frequency of active and inactive compounds) and for this amino acid, the mutational data consistently indicate a significant decrease in activity for all examined ligands. The mutation of L7x38 was extensively examined for different ligands and varying substitution schemes, leading to a slight decrease of affinity in the majority of cases and a lack of change in one case. The importance of the Y7x42 position is undeniable, as its mutation led to either a significant decrease in ligand affinity or a complete loss of activity. Position S5x43, which displayed high discriminative power in 7UM7-based docking studies, is related to inconsistent mutagenetic data – the influence on ligand affinity (increase/decrease) depended on the specific ligand and the substituted amino acid.

## Discussion

The conducted study on 5-HT_5A_R ligands has produced a wealth of constructive insights into the nature of this target. The results obtained have confirmed the significance of 5-HT_5A_R in drug design applications and provided valuable structural and interaction-based information for designing agents with the desired activity profile. The extensive range of reported compounds and their diverse structural characteristics and biological activity towards the target showcase the potential of 5-HT_5A_R in pharmacological research. The distribution of ligands between particular data sources highlights the importance of the conducted search and manual extraction of compound structures from patents. The most populated classes of 5-HT_5A_R ligands, that is acylguanidines, 2-aminoquinolines, and benzoxazines, which contained in total over 1500 records, were fetched exclusively from patents, which emphasizes the necessity of manual extraction for incorporating such compounds in the dataset. Despite quite significant structural diversity of the presented ligands, there are common features which were revealed by pharmacophore modeling, such as aromatic rings together with hydrophobic and donor moieties. The geometric relationships between these features vary for different chemical classes considered; however, there is a set of ligand–protein contacts, which are supposed to be formed in order to maximize the probability of 5-HT_5A_R activity. These interactions include contacts with D3x32, V3x33, C3x36, V45x52, S5x43, F6x51, and F6x52 amino acids, which were confirmed both in in silico studies (docking and molecular dynamics simulations), as well as in the experimental research (mutagenetic studies). The alignment of the docking outcome with the previously reported mutagenetic data confirms the reliability of the computational approaches applied. Further validation of the applied computational protocols was obtained through the correlational studies of the MD output with the in vitro reports. All the obtained results can be utilized in the design of agents with selective or non-selective activity profiles towards 5-HT_5A_R.

## Conclusions

In the study, we prepared a comprehensive collection of 2160 5-HT_5A_R ligands supplied with a detailed examination of their potential interactions with the target protein. The systematic description of the chemical groups of already known ligands, supplied with the pharmacophore modeling and in-depth analysis of docking results and MD simulation studies for selected ligands, enabled the determination of key factors to consider when designing new 5-HT_5A_R agents. By focusing on different chemical classes of 5-HT_5A_R ligands during modeling, we have enhanced the utility gained knowledge by capturing in detail the ligand–protein contacts characteristic for a given scaffold. The incorporation of different crystal structures and bioactivity data from various sources makes the study comprehensive and highly valuable for the medicinal chemistry community, providing guidance for successful development of compounds displaying activity towards 5-HT_5A_R. Furthermore, the MD-based analysis is supported by the correlation of the computational outcome with the biological results measured in vitro, which further validates our findings.

## Supplementary Information

Below is the link to the electronic supplementary material.

Supplementary file1 (DOCX 1174 KB)

Supplementary file2 (DOCX 31 KB)

Supplementary file3 (DOCX 615 KB)

Supplementary file4 (XLSX 125 KB) File S2. A collection of 5-HT_5A_R ligands covering literature and patent data

## Data Availability

The 5-HT_5A_R dataset prepared in the study is available in the Supplementary Information. Detailed molecular modeling outcomes are available from authors upon request.

## Abbreviations

5-CT: 5-Carboxamidotryptamine
5-HT_5A_R: 5-Hydroxytryptamine receptor
7TM: Seven transmembrane
GPCR: G protein-coupled receptor
MD: Molecular dynamics
SAR: Structure–activity relationship
SIFt: Structural interaction fingerprint
