# Supplementary material for: Uncovering the unique characteristics of different groups of 5-HT5AR ligands with reference to their interaction with the target protein
Source: Pharmacol Rep. 2024 Jul 6;76(5):1130–46. doi: 10.1007/s43440-024-00622-4 (PMC11387456; doi:10.1007/s43440-024-00622-4)
Supplement: Supplementary file 1 — Supplementary file1 (DOCX 1174 KB) [file 43440_2024_622_MOESM1_ESM.docx]

**
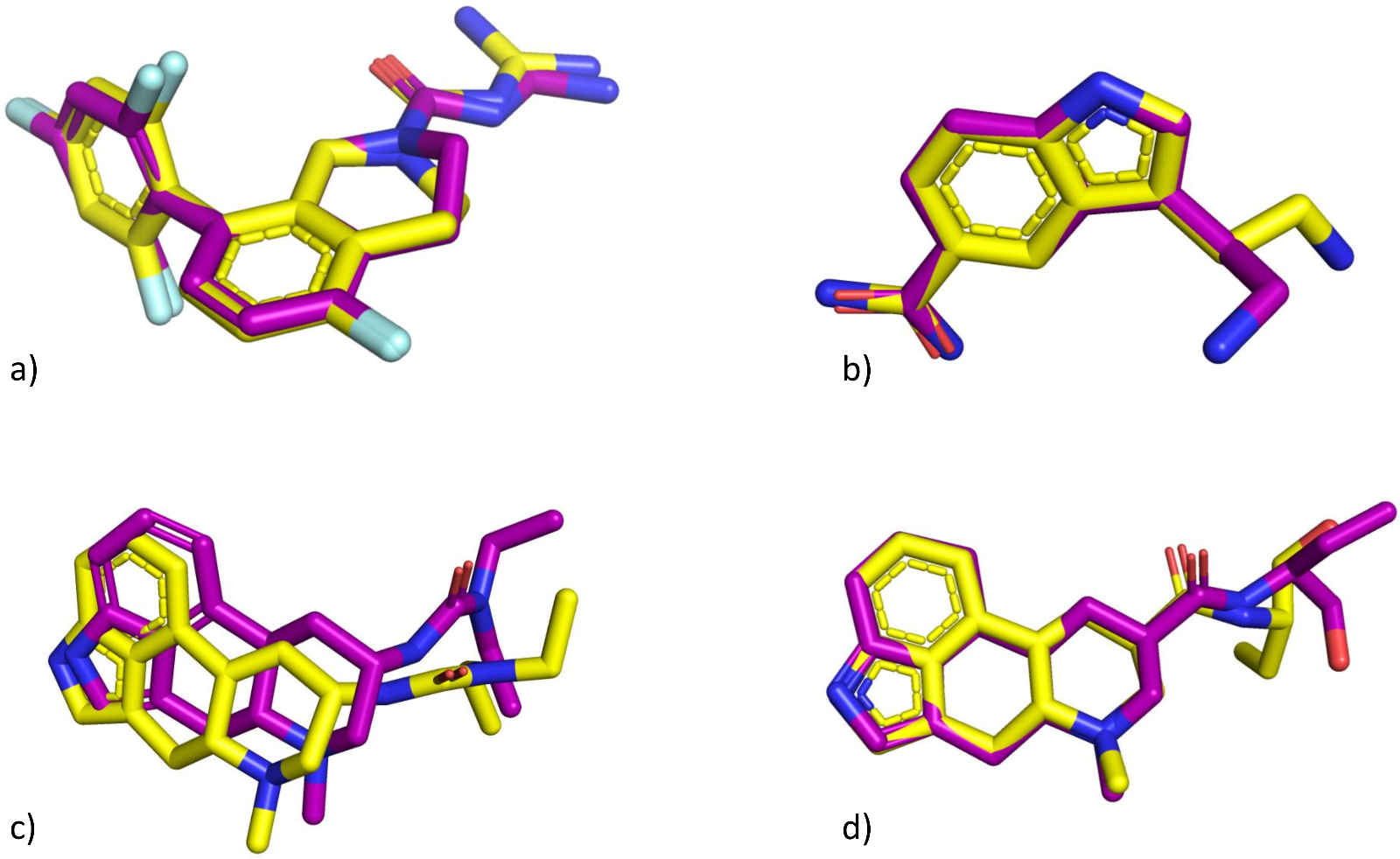
**

**Figure S1**. Comparison of the co-crystallized ligands (yellow) with their respective poses obtained in docking (purple) for a) 7UM4, b) 7UM5, c) 7UM6, d) 7UM7.

The figure presents the comparison of compound conformations obtaiend in docking (purple) with the co-crystallized orientations in the 5-HT_5A_R binding site.
