# Supplementary material for: Uncovering the unique characteristics of different groups of 5-HT5AR ligands with reference to their interaction with the target protein
Source: Pharmacol Rep. 2024 Jul 6;76(5):1130–46. doi: 10.1007/s43440-024-00622-4 (PMC11387456; doi:10.1007/s43440-024-00622-4)
Supplement: Supplementary file 2 — Supplementary file2 (DOCX 31 KB) [file 43440_2024_622_MOESM2_ESM.docx]

**Table S1**. Summary of particular clusters of 5-HT_5A_R ligands.

The subsequent columns contain the following pieces of information: the number of cluster, name of the cluster related to the leading chemical structure, the number of compounds belonging to the cluster, and information about the data source.

| **Cluster** | **Name** | **Size** | **Source** |
| --- | --- | --- | --- |
| 1 | Acylguanidines | 906 | Patents (Astella Pharma) [1–7] |
| 2 | 2-Aminoquinolines | 448 | Patents (Hoffmann La Roche) [8–15] |
| 3 | Benzoxazines | 172 | Patents (Hoffmann La Roche) [16,17] |
| 4 | 2-Amino-dihydroquinazolines | 153 | Patents (Hoffmann La Roche) [18–24], ChEMBL |
| 5 | N'‐benzyl‐N‐(pyridin‐2‐yl)guanidines | 97 | Patent (AbbVie Deutschland GmbH & Co. Kg) [25] |
| 6 | Benzimidazoles | 90 | Patent (Daiichi Sankyo Co Ltd) [26] |
| 7 | Biarylmethylamines | 59 | Patent (Glaxo Group Limited) [27], ChEMBL |
| 8 | Aminoimidazoles | 54 | Patent (AbbVie Deutschland GmbH & Co. Kg) [28] |
| 9 | Carbolines | 30 | ChEMBL, PDSP |
| 10 | Lysergic acid derivatives | 20 | ChEMBL, PDSP |
| 11 | Arylpiperazines | 20 | ChEMBL, PDSP |
| 12 | Isoquinoline amides | 20 | Patent (Astellas Pharma) [29] |
| 13 | 1‐(quinolin‐8‐yl)methanamines | 13 | ChEMBL |
| 14 | Quinazolines | 12 | Patent (Sumitomo Pharma Co Ltd) [30] |
| 15 | 2‐chloro‐2'‐methoxy‐1,1'‐biphenyls | 10 | ChEMBL |
| 16 | Tricyclic | 10 | ChEMBL, PDSP |
| 17 | Tryptamine derivatives | 9 | ChEMBL, PDSP |
| 18 | Arylpiperidines | 7 | ChEMBL, PDSP |
| 19 | 1,3,5‐triazine‐2,4‐diamines | 5 | ChEMBL |
| 20 | 1,2,3,4-tetrahydronaphthalen-1-ones | 5 | PDSP |
| 21 | aplysinopsin analogs | 5 | ChEMBL |
| 22 | 1‐{2‐[1‐(arylsulfonyl)pyrrolidin‐2‐yl]ethyl}piperidines | 4 | ChEMBL, PDSP |
| 23 | Diarylalkylamines | 4 | ChEMBL |
| 24 | 5-OH-DPAT analogs | 3 | ChEMBL |
| 25 | 1,2,3,4-Tetrahydroisoquinolines | 2 | ChEMBL |
| 26 | N‑Methyllaurotetanine | 1 | ChEMBL |
| 27 | Mellpaladine B | 1 | PDSP |

[1] Kinoyama I, Miyazaki T, Koganemaru Y, Shiraishi N, Kawamoto Y, Washio T. Acylguanidine derivative. WO2010090304A1, 2010.

[2] Kinoyama I, Koganemaru Y, Miyazaki T, Washio T. Substituted acylguanidine derivative. WO/2010/090305, 2010.

[3] Hamaguchi W, Kinoyama I, Koganemaru Y, Miyazaki T, Kaneko O, Sekioka R, et al. Tetrahydroisoquinoline derivative. US8962612, 2015.

[4] Kinoyama I, Satoshi M, Hiroaki H, Miya ZS, Mountain ZM. Acylguanidine derivatives. JP5287257, 2013.

[5] Kinoyama I, Miyamoto S, Miyazaki T, Koganemaru Y, Kawamoto Y, Shiraishi N, et al. Bicyclic acylguanidine derivatives. JP5569857, 2014.

[6] Kinoyama I, Miyazaki T, Koganemaru Y, Shiraishi N, Kawamoto Y, Washio T. Acylguanidine derivative. WO/2010/090304, 2010.

[7] Kinoyama I, Miyazaki T, Koganemaru Y, Washio T, Wataru H. Nitrogenous-ring acylguanidine derivative. WO/2011/016504, 2011.

[8] Kolczewski S, Riemer C, Roche O, Steward L, Wichmann J, Woltering T. 2-Aminoquinolines. WO/2009/109502, 2009.

[9] Kolczewski S, Riemer C, Roche O, Steward L, Wichmann J, Woltering T. 2-Aminoquinolines as 5-HT5A receptor antagonists. WO/2009/112395, 2009.

[10] Kolczewski S, Riemer C, Roche O, Steward L, Wichmann J, Woltering T. 2-Aminoquinoline derivatives. WO/2009/109491, 2009.

[11] Kolczewski S, Riemer C, Roche O, Steward L, Wichmann J, Woltering T. 2-Aminoquinolines. WO/2009/109477, 2009.

[12] Kolczewski S, Riemer C, Steward L, Wichmann J, Woltering T. Quinoline derivatives as 5HT5A receptor antagonists. WO/2009/040290, 2009.

[13] Kolczewski S, Riemer C, Steward L, Wichmann J, Woltering T. 2-Aminoquinolines as 5-HT(5A) receptor antagonists. WO/2008/068157, 2008.

[14] Kolczewski S, Riemer C, Steward L, Wichmann J, Woltering T (Grenzach-W. 2-Aminoquinoline derivatives. US7825253, 2010.

[15] Kolczewski S, Riemer C, Roche O, Steward L, Wichmann J, Woltering T. 2-Aminoquinoline derivatives as 5-HT(5A) receptor antagonists. WO/2009/109493, 2009.

[16] Kolczewski S, Roche O, Steward L, Wichmann J, Woltering T. 5-Substituted benzoxazines. WO/2010/026112, 2010.

[17] Kolczewski S, Roche O, Steward L, Wichmann J, Woltering T. 6-Substituted benzoxazines as 5-HT-5A receptor antagonists. WO/2010/026110, 2010.

[18] Alanine A, Gobbi LC, Kolczewski S, Luebbers T, Peters J-U, Steward L. Use Of 2-Anilino-3,4-dihydro-quinazolines as 5HT5A receptor antagonists. WO/2006/097391, 2006.

[19] Alanine A, Gobbi LC, Kolczewski S, Luebbers T, Peters J-U, Steward L. (3,4-Dihydro-quinazolin-2-yl)-indan-1-yl-amines. US7348332, 2008.

[20] Alanine A, Gobbi LC, Kolczewski S, Luebbers T, Peters J, Steward L. (3,4-Dihydro-quinazolin-2-yl)-(2-aryloxy-ethyl)-amines having an activity on the 5-HT receptor. WO/2006/117305, 2006.

[21] Alanine A, Gobbi LC, Kolczewski S, Luebbers T, Peters J, Steward L. 8-Alkoxy or cycloalkoxy-4-methyl-3,4-dihydro-quinazolin-2-ylamines. 20060293350, 2006.

[22] Alanine A, Gobbi LC, Kolczewski S, Luebbers T, Peters J, Steward L. 8-Alkoxy or cycloalkoxy-4-methyl-3,4-dihydro-quinazolin-2-ylamines. US7790733, 2010.

[23] Alanine A, Gobbi LC, Kolczewski S, Luebbers T, Peters J, Steward L. 5-Chloro-4-alkyl-3,4-dihydro-quinazolin-2-ylamine derivatives. US7790732, 2010.

[24] Alanine A, Gobbi LC, Kolczewski S, Luebbers T, Peters J, Steward L. 5-Chloro-4-alkyl-3,4-dihydro-quinazolin-2-ylamine derivatives. US20060293349, 2006.

[25] Amberg W, Netz A, Kling A, Ochse M, Lange U, Hutchins CW, et al. Hetaryl-substituted guanidine compounds and use thereof as binding partners for 5-HT5-receptors. US9296697, 2016.

[26] Suzuki R, Katayama K, Ueno S, Sugimoto Y, Watanabe H. Benzimidazole Derivative. WO/2020/095912, 2020.

[27] Bromidge SM, Corbett DF, Heightman TD, Moss SF. Biaryl Compounds Having Activity At The 5HT5A Receptor. WO/2004/096771, 2004.

[28] Amberg W, Netz A, Kling A, Ochse M, Lange U, Haupt A, et al. 5-Ring Heteroaromatic Compounds And Their Use As Binding Partners For 5-HT5 Receptors. US20150183770, 2015.

[29] Hamaguchi W, Koganemaru Y, Sekioka R, Kaneko O, Kato K. Isoquinoline Amide Derivative. JP2014076948, 2014.

[30] Tsuda Y, Fukaya T, Kitahara K, Nakai Y. Quinazolinone Derivative. JP2015124211, 2015.
