# Supplementary material for: Uncovering the unique characteristics of different groups of 5-HT5AR ligands with reference to their interaction with the target protein
Source: Pharmacol Rep. 2024 Jul 6;76(5):1130–46. doi: 10.1007/s43440-024-00622-4 (PMC11387456; doi:10.1007/s43440-024-00622-4)
Supplement: Supplementary file 3 — Supplementary file3 (DOCX 615 KB) [file 43440_2024_622_MOESM3_ESM.docx]

**History** **of 5-HT_5A_R ligands discoveries**

For a long time, only compounds with weak and non-selective binding to 5-HT_5A_R were known [1-7]. However, in 2003, the first robust screening against 5-HT_5A_R took place. It involved testing a series of tetrahydrocarboline derivatives on both human and mouse 5-HT_5A_ receptors. The results showed that the intact ring system (tetrahydro-γ-carbo-line or tetrahydro-β-carboline ring) was the most crucial factor for the 5-HT_5A_R activity. The most favorable compound displayed an affinity of 10 nM (K_i_) but also demonstrated strong affinity to 5-HT_2_ receptor, which is a typical characteristic of derivatives with the tetrahydrocarboline core. After further optimization, the 4-methoxy substituted compound and its ether version were obtained, both of which displayed a 4-fold preference for 5-HT_5A_R over 5-HT_2A_R [8], which constituted a valuable insight into the development of compounds that selectively target the 5-HT_5A_R.

In 2005, a high-throughput screening investigating various biphenylmethylamines culminated in the identification of a novel class of compounds characterized by pronounced binding affinity for the 5-HT_5A_R. Analysis of the structure-activity relationship (SAR) within this class of compounds led to the identification of a potent and highly specific antagonist for the 5-HT_5A_R (K_i_ = 6.3 nM). It is worth noting that this antagonist, named SB-699551-A, exhibited an exceptional selectivity towards the serotonin transporter and receptors 5-HT_1A_/_B_/_D_, 5-HT_2A_/_C_, and 5-HT_7_ [9, 10].

In 2008, Peters et al. synthesized a series of compounds based on the structure of
2-aminodihydroquinazolines. Through their research, it was discovered that these compounds function as dual ligands for both 5-HT_5A_ and 5-HT_7_ receptors. Compounds with the highest potency displayed strong binding to both 5-HT_5A_R and 5-HT_7_R with the simultaneous high selectivity (over 30-fold) over other related receptors [11]. Through further optimization and adjustments in lipophilicity and pK_a_ values, a 20-fold enhancement in the brain-to-plasma ratio was attained, resulting in micromolar concentrations of the compound in the brain following oral administration [12].

The years 2014 and 2015 saw two extensive screening campaigns being conducted, which yielded some promising results. Two compounds, namely US-8853242-B2 and US-8962612-B2, were found to exhibit the highest binding affinity to 5-HT_5A_R. In the former case, a set of derivatives of acylguanidine were synthesized, where the guanidine moiety was attached to the quinoline or isoquinoline ring through a carbonyl group. This resulted in eighteen antagonists, all of which displayed K_i_ values below 15 nM (the best compound was characterized by the K_i_ of 1.3 nM) [13, 14]. In the second patent, derivatives of tetrahydroisoquinoline were synthesized with an acylguanidine group attached through the N-atom of the aromatic ring. A total of 20 compounds were obtained with K_i_ values below 10 nM, and the most potent compound achieved an activity level of 0.68 nM [15].

In 2022, the docking of over 6 million molecules to 5-HT_5A_R led to the identification 5 new ligands of this receptor. Further investigation and optimization were carried out on one of these ligands, which resulted in the synthesis of a compound UCSF678. This compound was found to exhibit weak partial agonism activity and β-arrestin bias against the 5-HT_5A_R [16].

The summary of the 5-HT_5A_R ligands discoveries is presented in Figure 1.

**Figure 1**. History of the development of 5-HT_5A_R ligands.


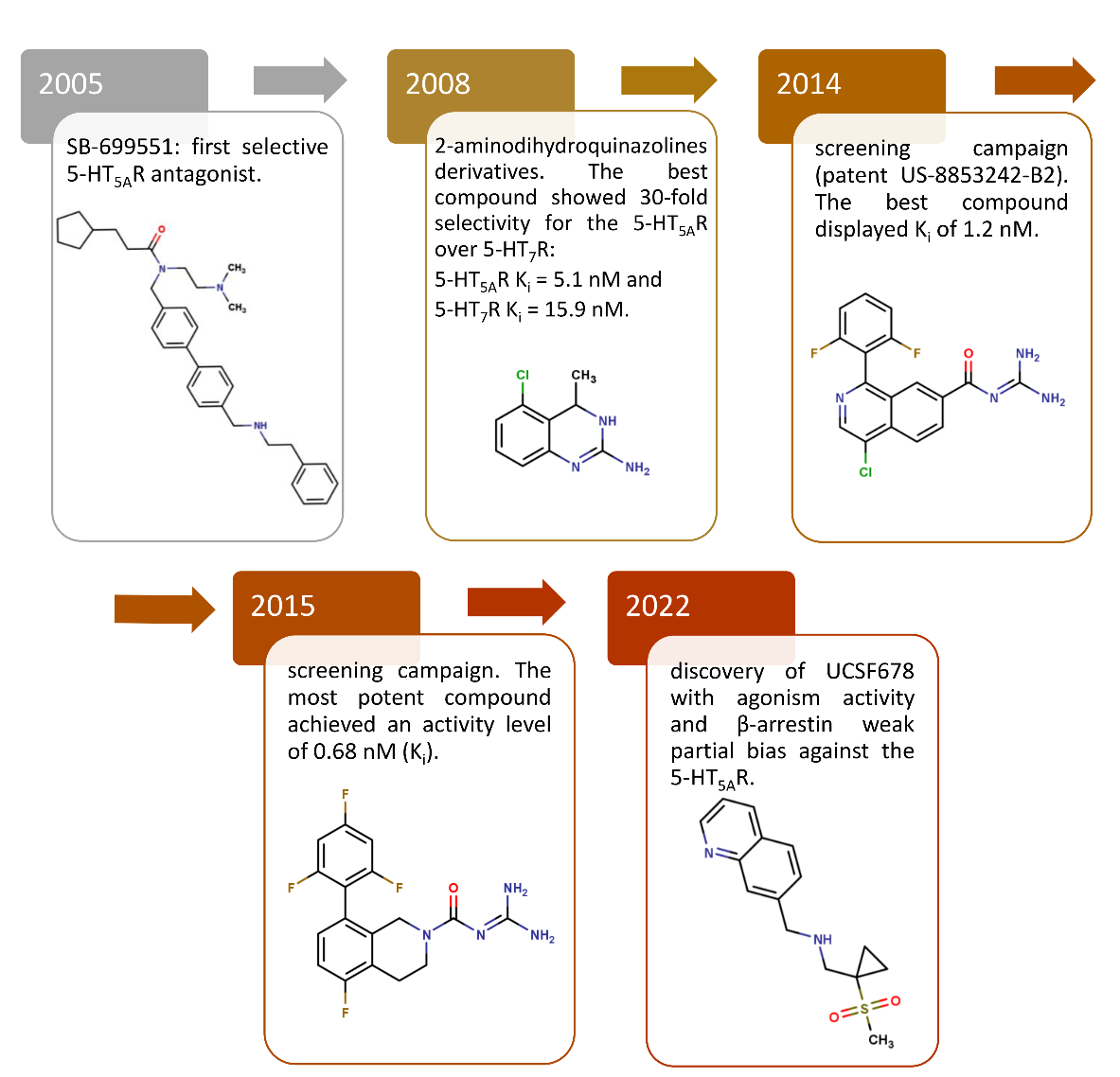


**References**

[1] Lovell PJ.; Bromidge SM, Dabbs S, Duckworth DM, Forbes IT, Jennings AJ et al. A Novel, Potent, and Selective 5-HT7 Antagonist: (R)-3-(2-(2-(4-Methylpiperidin-1-Yl)- Ethyl)Pyrrolidine-1-Sulfonyl)Phenol (SB-269970). J Med Chem 2000, 43(3): 342–345.

[2] Glennon RA. Higher-End Serotonin Receptors:  5-HT5, 5-HT6, and 5-HT7. J Med Chem 2003, 46(14): 2795–2812

[3] Poulain R, Horvath D, Bonnet B, Eckhoff C, Chapelain B, Bodinier MC, Déprez B. From Hit to Lead. Analyzing Structure-Profile Relationships. J Med Chem 2001, 44(21): 3391–3401.

[4] Nichols DE, Frescas S, Marona-Lewicka D, Kurrasch-Orbaugh DM. Lysergamides of Isomeric 2,4-Dimethylazetidines Map the Binding Orientation of the Diethylamide Moiety in the Potent Hallucinogenic Agent N,N-Diethyllysergamide (LSD). J Med Chem 2002, 45(19): 4344–4349.

[5] Ferretti G, Dukat M, Giannella M, Piergentili A, Pigini M, Quaglia W et al. Homoazanicotine: A Structure-Affinity Study for Nicotinic Acetylcholine (NACH) Receptor Binding. J Med Chem 2002, 45(21): 4724–4731.

[6] Willoughby CA, Hutchins SM, Rosauer KG, Dhar MJ, Chapman KT, Chicchi GG et al. Combinatorial Synthesis of 3-(Amidoalkyl) and 3-(Aminoalkyl)-2-Arylindole Derivatives: Discovery of Potent Ligands for a Variety of G-Protein Coupled Receptors. Bioorg Med Chem Lett 2002, 12(1): 93–96.

[7] Forbes IT, Douglas S, Gribble AD, Ife RJ, Lightfoot AP, Garner AE et al. SB-656104-A: A Novel 5-HT7 Receptor Antagonist with Improved in Vivo Properties. Bioorg Med Chem Lett 2002, 12(22): 3341–3344.

[8] Khorana N, Smith C, Herrick-Davis K, Purohit A, Teitler M, Grella B et al. Binding of tetrahydrocarboline derivatives at human 5-HT5A receptors. J Med Chem. 2003, 46(18): 3930-3937.

[9] Corbett DF, Heightman TD, Moss SF, Bromidge SM, Coggon SA, Longley MJ et al. Discovery of a potent and selective 5-ht5A receptor antagonist by high-throughput chemistry. Bioorg. Med. Chem. Lett. 2005,15(18): 4014-4018.

[10] Thomas DR, Soffin EM, Roberts C, Kew JN, de la Flor RM, Dawson LA, Fry VA et al. SB-699551-A (3-cyclopentyl-N-[2-(dimethylamino)ethyl]-N-[(4'-{[(2-phenylethyl)amino]methyl}-4-biphenylyl)methyl]propanamide dihydrochloride), a novel 5-ht5A receptor-selective antagonist, enhances 5-HT neuronal function: Evidence for an autoreceptor role for the 5-ht5A receptor in guinea pig brain. Neuropharmacology. 2006, 51(3): 566-577.

[11] Peters JU, Lübbers T, Alanine A, Kolczewski S, Blasco F, Steward L. Cyclic guanidines as dual 5-HT5A/5-HT7 receptor ligands: optimising brain penetration. Bioorg Med Chem Lett. 2008, 18(1): 262-266.

[12] Peters JU, Lübbers T, Alanine A, Kolczewski S, Blasco F, Steward L. Cyclic guanidines as dual 5-HT5A/5-HT7 receptor ligands: structure-activity relationship elucidation. Bioorg Med Chem Lett. 2008, 18(1): 256-261.

[13] Yamazaki M, Harada K, Yamamoto N, Yarimizu J, Okabe M, Shimada T, Ni K, Matsuoka N. ASP5736, a novel 5-HT5A receptor antagonist, ameliorates positive symptoms and cognitive impairment in animal models of schizophrenia. Eur Neuropsychopharmacol. 20142, 24(10): 1698-1708.

[14] Kinoyama I, Miyazaki T, Koganemaru Y, Washio T, Hamaguchi W. US-8853242-B2, 2014.

[15] Hamaguchi W, Kinohama I, Koganemaru Y, Miyazaki T, Kaneko O, Sekioka R, Washio T. US-8962612-B2, 2015.

[16] Levit Kaplan A, Strachan RT, Braz JM, Craik V, Slocum S, Mangano T et al. Structure-Based Design of a Chemical Probe Set for the 5-HT_5A_ Serotonin Receptor. J Med Chem. 2022, 65(5): 4201-4217.
